# Generalist dispersers promote germination of an alien fleshy-fruited tree invading natural grasslands

**DOI:** 10.1371/journal.pone.0172423

**Published:** 2017-02-16

**Authors:** Martín Raúl Amodeo, María Belén Vázquez, Sergio Martín Zalba

**Affiliations:** 1 GEKKO (Grupo de Estudios en Conservación y Manejo), Departamento de Biología, Bioquímica y Farmacia, Universidad Nacional del Sur - CONICET, Bahía Blanca, Argentina; 2 CERZOS (Centro de Recursos Renovables de la Zona Semiárida), Centro Científico Tecnológico Bahía Blanca, CONICET, Bahía Blanca, Argentina; Consejo Superior de Investigaciones Cientificas, SPAIN

## Abstract

Plants with animal-dispersed fruits seem to overcome the barriers that limit their spread into new habitats more easily than other invasive plants and, at the same time, they pose special difficulties for containment, control or eradication. The effects of animals on plant propagules can be very diverse, with positive, neutral or negative consequences for germination and recruitment. Moreover, the environmental conditions where the seeds are deposited and where the post-dispersal processes take place can be crucial for their fate. *Prunus mahaleb* is a fleshy-fruited tree invading natural grasslands in the Argentine Pampas. In this study, we analyzed the importance of pulp removal, endocarp scarification and the effects of vectors on its germination response, by means of germination experiments both in the laboratory and under semi-natural conditions. Our laboratory results demonstrated that endocarp scarification enhances germination and suggests that vestiges of pulp on the stones have inhibitory effects. Frugivores exert a variety of effects on germination responses and this variation can be explained by their differing influence on pulp removal and endocarp scarification. Most frugivores produced a positive effect on germination under laboratory conditions, in comparison to intact fruits and hand-peeled stones. We observed different degrees of pulp removal from the surface of the stones by the dispersers which was directly correlated to the germination response. On the other hand, all the treatments showed high germination responses under semi-natural conditions suggesting that post-dispersal processes, like seed burial, and the exposure to natural conditions might exert a positive effect on germination response, attenuating the plant's dependence on the dispersers’ gut treatment. Our results highlight the need to consider the whole seed dispersal process and the value of combining laboratory and field tests.

## Introduction

Invasive species constitute a central component of global change, as well as other novel organisms (range-expanding species, genetically modified organisms, synthetic organisms, emerging pathogens) [1]. The movement of species into new habitats offers an opportunity to test hypotheses about the importance of certain biological processes, like the role of mutualistic interactions in the dispersal of plants. An introduced plant faces several barriers to colonizing a new environment, and many of these species rely on mutualistic interactions with local species [2,3]. Plants with animal-dispersed fruits seem to overcome the barriers that limit their spread into new habitats more easily than other invasive plants [4–6]. Many of these species maintain diffuse interactions with a range of dispersal agents in their native ranges, and the life-history traits associated with this behavior may play a key role for facilitating the establishment of novel dispersal mutualisms in invaded regions [4,7–10]. The management of invasive fleshy-fruited plants is a main challenge for biodiversity conservation [4,5,11]. A better understanding about the role of generalist frugivores in invasive plant dispersal is crucial for orientating management decisions.

The seeds of endozoochoric plants may have various complex mechanisms of dormancy that modulate their germination, involving physiological, morphological and physical factors [12], which can be released by certain environmental signals [13]. On the other hand, the behavior of frugivores can be diverse and hence, their effects on the seed during ingestion, processing and defecation, can have favorable, neutral or negative consequences for germination [14,15]. Effective seed dispersal depends on both plant traits and frugivore characteristics, such as behavior, body size, home range, seed passage time and digestive physiology, which all affect the probability of a dispersed seed becoming a new reproductive adult in the population [16]. The treatment given by frugivores to fruits include the total or partial separation of pulp and seed, the scarification of the seed or endocarp, and the environmental conditions of the microhabitat where they are deposited [11,15]. The seed may be transported to distances far from the zone of influence of the parent plant during treatment inside the gut, reducing the effects of host-specific seed predators, herbivores and pathogens which act in a density- and/or distance-dependent manner [17]. The environmental conditions and post-dispersal processes at deposition sites, such as secondary dispersal or natural burial mechanisms, may also have an important role on germination and establishment [15,18,19]. Moreover, soil processes, like the effect of microorganisms and environmental fluctuations, have been highlighted as key factors affecting seed fate and recruitment [11,20,21].

The production and survival of seeds, together with their germination capacity, have been identified as key components contributing to the expansion of fleshy-fruited invasive plants [22], which often have larger crop sizes and/or more attractive fruits than native plants [7]. Moreover, they have been shown to be effectively dispersed by birds and mammals, often exhibiting higher germination rates after seed consumption by frugivores [5]. Most of the available information about the dispersers’ effects on germination comes from studies that use a single methodological approach, only testing the effects of frugivores under one experimental condition, mostly in the laboratory [23,24]. The conditions under which experiments are undertaken may obscure the differences between treatments as both laboratory and field experiments are important. The former help to isolate factors and the latter allow the replication of the natural conditions. Hence the study of the effects of frugivores on seed germination must combine different experimental conditions in order to fully understand the dispersers’ effects on seed germination [15,23–25].

The proliferation of invasive trees and shrubs in natural grasslands has been one of the most striking land cover changes worldwide over the past century, driving major consequences for biodiversity and ecosystem functioning [26]. Natural grasslands in the South American Pampas have suffered severe alterations and only a small proportion of the original surface is included in protected areas [27,28]. A set of invasive woody plants of diverse origins have spread over the native biodiversity in the last remnants of natural grasslands in the Pampas eco-region [29], among them, St. Lucie’s cherry (*Prunus mahaleb*) has established dispersal interactions with local fauna benefiting from their dispersal service [30,31]. In spite of the profuse work on the interactions between this species and the seed dispersers within its native distribution [32–34], there are no reports about the effects of seed consumers on its germination response. At least 28 bird species, four mammals and one lizard have been recorded feeding on its fruits within its native range of distribution [33,35]. In our study area, we recorded 20 birds, seven mammals and one ant species manipulating its fruits [31]. In this paper, we study its germination response in invasive populations expanding over natural grasslands in the Argentine Pampas. We evaluate the importance of pulp removal and mechanical and chemical endocarp scarification, analyzing the effects that different vectors have on seed germination and we compare germination responses under different experimental conditions during a two-year-study. We propose that *P*. *mahaleb* benefits from the treatment of generalist vectors that promote germination through pulp removal and endocarp scarification.

## Materials and methods

### Study species

*Prunus mahaleb* L. is a small fleshy-fruited tree native to Eurasia, frequently used as a rootstock for orchard trees, as an ornamental plant and also for timber production [36,37]. These have been the underlying reasons for its introduction and invasion in several countries, such as the United States [38], Canada [39], Australia [36], New Zealand [40] and Argentina [30]. The production and ripening of fleshy fruits take place during approximately two months in late spring and early summer, both in Europe and in the Argentine Pampas [41,42]. One-seeded drupes are held in clusters of one to ten fruits, bright black when ripe, with a diameter of eight millimeters and a pulp-seed ratio of 2.44:1 in volume and 3.85:1 in wet mass [31]. The fruits have a sugary water-rich pulp [32].

There are various degrees of physiological dormancy in *Prunus* species combining chemical inhibitors in the embryo and mechanical resistance to germination by the endocarp [37,43]. Different methods have been employed successfully to break embryo dormancy in *P*. *mahaleb*, including cold stratification or exposure to gibberellins [44–46]. All *Prunus* species are characterized by having a hard endocarp offering a certain mechanical resistance to germination, but being permeable to water [12,37,43]. Some species require scarification treatment but in other cases the growing embryo is strong enough to break the endocarp and germinate, once embryo dormancy is neutralized by stratification [37,43,47]. In *P*. *mahaleb*, the mechanical removal or weakening of the endocarp has been shown to enhance germination, while chemical scarification has led to different results, showing positive and negative effects [45,46,48]. Pulp and fruit extracts have been reported to have inhibitory effects on seed germination for other cherries [20,49,50]. Here we present the first study of fruit pulp effects in *P*. *mahaleb*.

### Study area

This study was carried out at the Ernesto Tornquist Provincial Park (38° 3.90’ S; 61° 58.33’ W), a nature reserve covering ca. 6700 ha of mountain grasslands in the southern Argentine Pampas. Access and work permits were issued by the Provincial Agency for Sustainable Development (OPDS), Buenos Aires province. This reserve is recognized as one of the few protected areas in the whole eco-region that includes remnant grasslands with a good conservation status [28,29,51]. The climate is temperate semi-arid and the vegetation is dominated by grass steppe (including *Stipa* spp., *Nassella* spp., *Piptochaetium* spp., *Festuca* ssp. and *Briza* spp.) [51,52] with exotic woody plants growing in small plantations and as spontaneous stands or isolated individuals [29]. A small number of native species with fleshy fruits can be found scattered in the area, such as *Cereus spp*., *Geoffroea decorticans* and *Margyricarpus pinnatus*, but their dispersal interactions have not been studied [52]. Among the exotic woody plants there are some fleshy-fruited shrubs, such as *Rosa sp*. and *Rubus ulmifolius*, which have a restricted distribution and low abundance [29]. *P*. *mahaleb* populations are however commonly found in valleys and on hillsides. The densest populations are associated with streams and canyons where dispersers’ activity seems to be higher [30,31], and smaller stands can also be found at mid-elevation on the hillsides.

### Seed collection

During the summer of 2010–2011, we collected ca. 1500 mature fruits of *P*. *mahaleb* directly from the branches of 16 hermaphrodite trees (height: 2–5 m, basal stem diameter: 25–50 cm, population 1). Hermaphrodite trees were selected because that is the most abundant gender type at the study site, although no differences have been found in germination rates between gender types [31]. After collection, approximately 300 intact fruits were spread out in a container and left to dry. The pulp (exocarp and mesocarp) of another set of 800 fruits was removed manually under running water. The resulting clean stones were left to dry in a container and this set was used for three treatments: mechanical scarification, chemical scarification, and hand-peeled stones. Both intact fruits and hand-peeled stones were used as control treatments following the recommendations of Samuels and Levey [23]. In order to obtain a better representation of the potential variability between populations, we collected a supplementary set of 200 hand-peeled stones from a different group of ten trees (height: 1–5 m, basal stem diameter: 10–40 cm, population 2) which was used as a supplementary control treatment in the laboratory. We use the term “stone” to refer to the true seed plus its endocarp.

Fruits consumed by dispersers were obtained by careful inspection and identification of droppings in the field during the summer of 2010–2011. We recorded the number of stones per dropping and collected the stones found in recently egested feces (less than 5 days old, defined by their desiccation) corresponding to the Pampas fox (*Lycalopex gymnocercus*), Spot-winged Pigeon (*Patagioenas maculosa*), Picazuro Pigeon (*Patagioenas picazuro*), Chalk-browed Mockingbird (*Mimus saturninus*), and also stones regurgitated by the Great Kiskadee (*Pitangus sulphuratus*) and Fork-tailed Flycatcher (*Tyrannus savana*). A group of stones was also collected from the entrance and refuse dumps of five different nests of the Black Leafcutter Ant (*Acromyrmex lundi*). The stones treated by dispersers were analyzed in order to assess the magnitude of pulp removal. For this purpose, 60 stones were randomly selected within each group (in the case of *A*. *lundi*, the 60 stones were collected evenly from three of the nests studied), estimating the percentage of its surface covered with pulp vestiges for each stone by careful visual inspection (using five categories: 0%, under 25%, 25–50%, 50–75%, above 75%).

After collection and drying, intact fruits, hand-peeled stones and the stones treated by dispersers were stored in paper bags in a dark dry place at room temperature for 88 days until the start of the experimental procedures. All the stones were stored until an appropriate date for sowing in the field, in order to ensure that the seeds used in laboratory test and in the experimental garden were all exposed to equal storage conditions and times. Based on previous experiments, this method of storage does not reduce the seed viability and germination response for the amount of time considered. All the groups of stones were randomly divided into two sets for the germination tests in the laboratory and experimental garden.

### Laboratory test

A germination experiment was carried out under laboratory conditions to compare the germination rates of 11 treatments: intact fruits, hand-peeled stones (from the two different populations), peeled and artificially-scarified stones, and those treated by dispersers. We used hand-peeled stones (population 1) for applying two scarification treatments: chemical and mechanical. The chemical scarification consisted of submerging the stones in 96% sulfuric acid for 10 minutes and then rinsing them under running water for 10 hours. Mechanical scarification was done by totally or partially removing the endocarp with a knife. After applying the treatments, all the stones were submerged in water for 24 hours and then placed in cold stratification (4–6°C) for 80–100 days [37,46]. The stones were kept in a humid sand substrate (1:3, stones and sand) in a plastic container which was watered regularly. Stones belonging to different treatments were separated in cloth bags.

After approximately 100 days of cold stratification the stones were transferred to a germination chamber under controlled temperature, photoperiod and humidity (14 hours light at 20°C / 10 hours dark at 10°C, RH = 50–70%). The experimental conditions were based on Prada and Arizpe [42] and Grisez *et al*. [37]. Stones were sown in plastic trays, in five replicates of 20 stones each per treatment. The substrate was cottonwool and sand [53] and stones were treated with zinc ethylenebisdithiocarbamate fungicide powder [54]. Counts were done every two to three days and germination was considered to have occurred when the primary root had emerged 2 mm outside the stone. The experiment was monitored every two days for 68 days: 20 days during the end of stratification and 48 days in the germination chamber.

At the end of the germination experiment, a viability test was carried out on the seeds that had not germinated. Stones were opened with a knife, extracting the endocarp and manually removing the seed coat. Both cotyledons were separated, only using the one that was adhered to the plumule and primary root. These were placed in a solution of 0.03% triphenyl tetrazolium chloride for 18–24 hours under dark conditions at 20°C. A seed was considered viable when the plumule or primary root and at least a portion of the cotyledon became stained. The percentage of viable seeds was calculated for each replicate, which contained between five and ten non-germinated stones. The average percentage was calculated for each treatment using the values from 4–5 replicates.

### Experimental garden

The emergence of seedlings was evaluated in an experiment under semi-natural conditions during the same year, which was carried out at the *Jardín Botánico Pillahuinco* (Ernesto Tornquist Provincial Park) using six treatments: intact fruits, hand-peeled stones, stones found in recent feces (less than five days old) corresponding to the Pampas fox (*L*. *gymnocercus*), Spot-winged Pigeon (*P*. *maculosa*), and stones regurgitated by the Great Kiskadee (*P*. *sulphuratus*) and Fork-tailed Flycatcher (*T*. *savana*). A total of 870 stones were sown: 150 per treatment in ten replicates of 15 stones each, except for *T*. *savana* for which we sowed 120 in eight replicates of 15 stones each. Intact fruits and hand-peeled stones were used as controls. In this case, 85 days after fruit collection (April 2011), the stones were sown individually in paper cylinders directly into the soil to a depth of 1 cm. The seed bed (1.2 m x 6 m) was covered with a transparent 200 micron nylon and a 50% shade-cloth so that solar radiation was attenuated and the rain effect was avoided, allowing a strict control over the irrigation regime. We irrigated homogenously over the seed bed once a week with a watering can, so that the quantity of water provided every month was equivalent to the median of precipitation value for each month corresponding to the historical records in the area for the period 1993–2009 (S1 Table, data provided by Ernesto Tornquist Provincial Park rangers and staff). The stones remained in the soil during the winter in order to replicate the natural stratification process, until germination started in spring. During spring, we recorded seedling emergence over a month until emergence stopped. Seedling survival and new seedling emergences were recorded during the spring of the following year. After that, the whole experiment was dismantled and all plants were uprooted.

### Data analysis

The final germination percentage and seedling emergence and survival were analyzed by means of the Generalized Linear Models with binomial distribution and *logit* link function [55]. Time to first germination and time to 50% of total germinated seeds were calculated in reference to the start of stratification process for both experiments. These temporal variables were analyzed using Generalized Linear Models with Gamma distribution and *log* link function. The effects of different treatments were compared using deviance ratio tests and Tukey tests for pair-wise comparisons. We used Spearman correlation analysis to assess the relationship between the final germination percentage in laboratory tests and the median of the percentage of stone surface covered with pulp vestiges (estimated using the categories as explained previously) for stones treated by *L*. *gymnocarcus*, *P*. *maculosa*, *M*. *saturninus*, *P*. *sulphuratus*, *T*. *savana* and *A*. *lundi*. Data analysis and graphics were carried out with R using packages *stats* [56], *multcomp* [57], *gridExtra* [58] and *ggplot2* [59].

## Results

### Laboratory test

**Fig 1:**
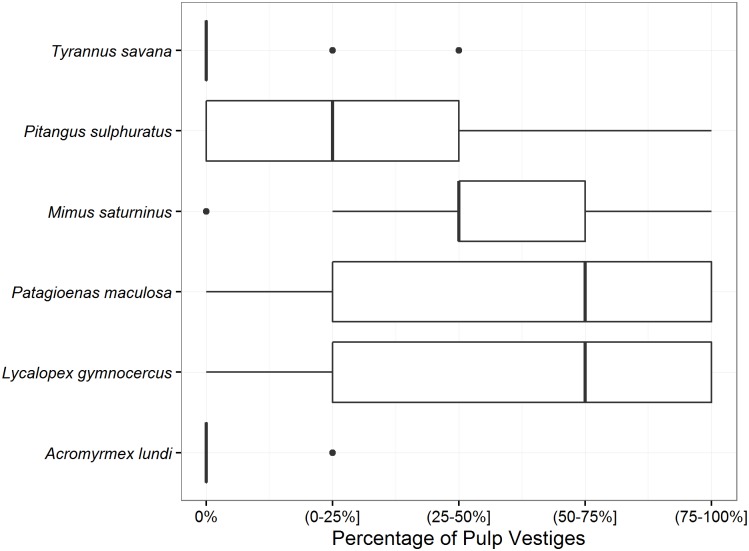
Percentage of stone surface covered with pulp vestiges for the *Prunus mahaleb* stones collected at the Ernesto Tornquist Provincial Park and exposed to treatment by animal dispersers. The percentage was estimated visually from a randomly selected sample of 60 stones for each disperser, using five categories of percentages. Box plots show the range, quartiles and medians for these categories.

The number of stones per dropping showed great variation between dispersers (Table 1), as well as the color and retention of pulp vestiges on the surface of the stones (Fig 1, S1 Fig). Most of the stones contained in the *P*. *picazuro* feces were destroyed (only one contained three undamaged stones out of a total of 27 analyzed feces).

**Table 1:** Number of stones per dropping and retention of pulp vestiges on the surface of stones of *Prunus mahaleb* collected from animal droppings at the Ernesto Tornquist Provincial Park, Argentina.

Species	Number of stones per dropping		
	Mean	Range	n
*P*. *maculosa*	4.03	[1–14]	76
*M*. *saturninus*	1.8	[1–5]	67
*P*. *sulphuratus*	8.63	[3–14]	34
*T*. *savana*	1	1	247
*L*. *gymnocercus*	161	[93–291]	10
*A*. *lundi* (nests)	-	[50–500]	5

In the laboratory, the final germination percentage differed significantly between the stones treated by different dispersers, as well as compared to those exposed to artificial scarification (Likelihood Ratio Test, Dev. = 47.83, d.f. = 48, p<0.0001, Table 2). After 85–138 days of cold stratification, the stones started to germinate showing variation between treatments. Those treatments with the highest germination percentage tended to germinate earlier and faster than those with a lower germination percentage. No signs of germination were observed in the intact fruits, whereas the hand-peeled stones showed low germination values. The maximum germination rate was reached by the stones treated by ants, which was almost twenty times greater than in the hand-peeled stones. All the stones consumed by birds showed a high germination rate, except for *P*. *maculosa*. The maximum value among these corresponded to the stones regurgitated by *T*. *savana* which was fifteen times higher than that observed for the hand-peeled stones, while values for stones regurgitated by *P*. *sulphuratus* and those found in feces of *M*. *saturninus* were twice to four times greater, respectively. The germination percentages of stones found in feces of *L*. *gymnocercus* and *P*. *maculosa* did not differ significantly from either the hand-peeled stones or the intact fruits (Table 2).

**Table 2:** Final germination percentage, time to first germination and time to 50% of total germination of *Prunus mahaleb* stones collected at the Ernesto Tornquist Provincial Park and exposed to different treatments under laboratory conditions: Intact fruits, hand-peeled stones, artificial scarification and treated by ants, birds, and foxes.

	Final Germination Percentage				Time to first germination		Time to 50% germination	
Treatment	Mean (%)	SE (%)	Odds Ratio	OR CI_95%_	Mean (Days)	SE	Mean (Days)	SE
Intact Fruits	0^a^	-	-	-	-	-	-	-
*Patagioenas maculosa* (Spot-winged Pigeon)	4^a^	1	0.34	[0.090–1.025]	113.0^c^	8.6	113.0^c^	8.6
*Lycalopex gymnocercus* (Pampas fox)	6^a^	3.6	0.52	[0.171–1.416]	110.7^c^	8.1	111.7^bc^	7.2
Hand-peeled stones (population 1)	11^ab^	3.7	0.12	[0.062–0.221]	93.2^ab^	1.7	94.0^a^	1.4
Hand-peeled stones (population 2)	15^ac^	3.5	1.43	[0.624–3.357]	93.8^ab^	3.0	94.8^ab^	2.9
*Pitangus sulphuratus* (Great Kiskadee)	23^bcd^	2.5	2.42	[1.129–5.452]	99.0^bc^	1.4	103.0^ac^	1.4
*Mimus saturninus* (Chalk-browed Mockingbird)	32^cd^	5.4	3.81	[1.838–8.412]	94.4^ab^	2.3	100.8^ac^	1.4
Chemical Scarification	36^d^	4.5	4.55	[2.216–10.004]	85.0^a^	-	96.2^ab^	2.8
*Tyrannus savana* (Fork-tailed Flycatcher)	65^e^	3.9	15.03	[7.347–33.201]	85.8^a^	0.8	98.4^ac^	0.6
Mechanical Scarifcation	66^e^	6.7	15.71	[7.669–34.755]	85.0^a^	-	91.0^a^	2.5
*Acromyrmex lundi* (leafcutter ant)	71^e^	4.6	19.81	[9.581–44.297]	85.0^a^	-	91.0^a^	2.5

Figures not sharing the same letters in the same column differ significantly at the 0.05 level of probability according to Tukey tests for pair-wise comparisons. The odds ratio for a binomial distribution modeling the final germination percentage are shown for the comparisons in reference to the hand-peeled stones (population 1), as well as the limits of the confidence interval (95%), obtained by likelihood profiles. Time to first germination and time to 50% of total germination were calculated in reference to the start of the stratification process, and were analyzed using Generalized Linear Models with Gamma distribution.

**Fig 2:**
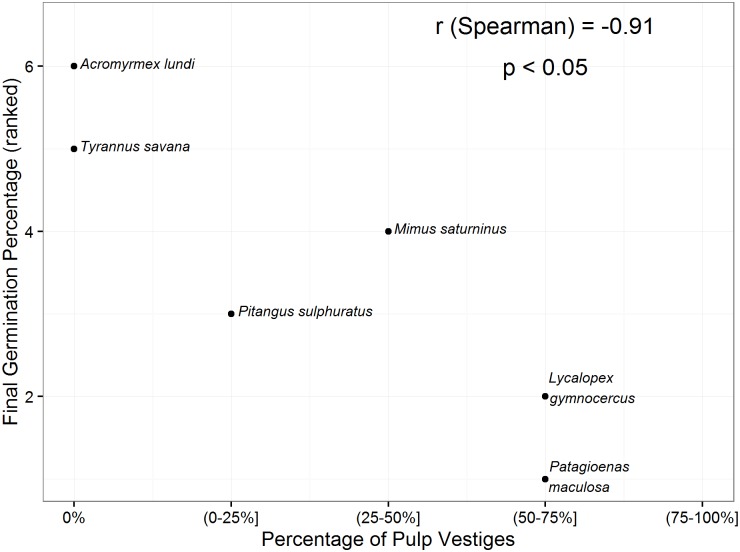
Relationship between the final germination percentage and the proportion of stone surface covered with pulp vestiges for *Prunus mahaleb* treated by different dispersers. The results of Spearman Correlation Analysis of the ranked variables are shown.

In the stones treated by animals, we found a significant negative relationship between the germination rate observed in the laboratory experiment and their median percentage of pulp cover (Spearman correlation, r = -0.91, S = 66.926, d.f. = 4, p = 0.011, Fig 2).

After 48 days of the germination experiment, the viability of non-germinated stones was still high for most of the treatments: 70% (SE = 6%, n = 5) for hand-peeled stones, 81% (SE = 2.6%, n = 4) for chemical scarification, 100% (n = 4) for *T*. *savana*, 87% (SE = 8.9%, n = 4) for *M*. *saturninus*, 85% (SE = 5.9, n = 4) for *P*. *sulphuratus*, 72% (SE = 7.2, n = 4) for *P*. *maculosa*, 62% (SE = 8.3%, n = 4) for *L*. *gymnocercus*.

### Experimental garden

In the experimental garden, the seedlings started to emerge between 146 and 173 days after sowing, and the time to the first seedling emergence did not vary significantly between the treatments. The 50% seedling emergence was reached between 166 and 186 days, and varied between treatments (Table 3). The final emergence percentage showed significant variation with respect to the treatments (*Likelihood Ratio Test*, Dev. = 49.86, d.f. = 864, p<0.001, Table 3), giving different results with respect to those obtained in the laboratory. Intact fruits and hand-peeled stones showed similar emergence rates, both exhibiting enhancements of 52% and 44% respectively with respect to those obtained in the laboratory. The emergence rate of stones regurgitated by *T*. *savana* was significantly higher with respect to the control treatments, in both hand-peeled stones and intact fruits, representing a 35% enhancement compared to what we found in the laboratory test. The emergence rate of stones consumed by *P*. *maculosa*, *P*. *sulphuratus* and *L*. *gymnocercus* did not differ significantly from that observed in the control treatments, but these values were 54%, 45% and 61% higher, respectively, than those obtained in the laboratory.

**Table 3:** Final seedling emergence percentage, time to first emergence and time to 50% of total emergence of *Prunus mahaleb* stones collected at the Ernesto Tornquist Provincial Park and exposed to different treatments in an experimental garden: Intact fruits, hand-peeled stones and treated by birds and foxes.

	Seedling Emergence		Time to First Emergence		Time to 50% Emergence	
Treatment	Mean (%)	SE (%)	Mean (Days)	SE	Mean (Days)	SE
Intact fruits	52^a^	4.1	157.7^a^	3.1	170.9^ab^	1.1
Hand-peeled stones	54.7^a^	4.1	161.7^a^	2.0	176.8^b^	2.6
*Patagioenas maculosa*	58^a^	4.1	157.0^a^	2.8	168.5^a^	1.8
*Lycalopex gymnocercus*	67.3^a^	3.8	153.0^a^	2.1	168.8^a^	1.1
*Pitangus sulphuratus*	68^a^	3.8	160.7^a^	2.5	173.5^ab^	2.3
*Tyrannus savana*	86.7^b^	3.1	156.0^a^	-	173.0^ab^	-

Seedling emergence was analyzed by means of Generalized Linear Models with binomial distribution and *logit* link function. Time to first emergence and time to 50% of total emerged seedlings were calculated in reference to the start of stratification and were analyzed using Generalized Linear Models with Gamma distribution and *log* link function. Figures not sharing the same letters in the same column differ significantly at the 0.05 level of probability according to the Tukey test for pair-wise comparisons.

Some of the total number of stones initially sown that did not germinate during the first year continued emerging during the second spring (2012), showing no significant differences between treatments (*Likelihood Ratio Test*, Dev. = 8.14, d.f. = 894, p = 0.14): 6% (SE = 2.6%) for intact fruits, 3.5% (SE = 2%) for hand-peeled stones, 0.9% (SE = 0.9%) for *T*. *savana*, 4.6% (SE = 2%) for *P*. *sulphuratus*, 6.4% (SE = 2.5%) for *P*. *maculosa*, 7.3% (SE = 2.5%) for *L*. *gymnocercus*. Seedlings that emerged during the first spring showed a high survival rate after one year (above 65%). Seedlings originating from hand-peeled stones presented a slightly higher survival rate than those consumed by some of the dispersers (Table 4).

**Table 4:** Survival rates for one-year-old *Prunus mahaleb* seedlings in the experimental garden at the Ernesto Tornquist Provincial Park, Argentina.

Treatment	Survival rate (%)	SE (%)
Intact fruits	84.3^ac^	3.9
Hand-peeled stones	91.7^c^	2.9
*Tyrannus savana*	83.8^bc^	3.5
*Pitangus sulphuratus*	71.9^ab^	4.3
*Patagioenas maculosa*	65.6^a^	4.9
*Lycalopex gymnocercus*	82.6^ac^	3.6

Seedling survival was analyzed by means of the Generalized Linear Models with binomial distribution and *logit* link function. Figures not sharing the same letters in the same column differ significantly at the 0.05 level of probability according to the Tukey test for pair-wise comparisons.

## Discussion

### Physical effects

In the laboratory, intact fruits did not show any signs of germination and, although pulp removal resulted in germination, the hand-peeled stones showed low germination rates demonstrating that the endocarp plays an important role in the dormancy of *P*. *mahaleb* seeds. Our results suggest that the pulp might have an inhibitory effect on germination, as has been reported for other *Prunus* species [15,20,49,50] and other invasive fleshy-fruited plants [5,11,60]. The pulp can inhibit germination by blocking the light or gas exchange, due to the presence of secondary metabolites that specifically inhibit certain enzymes involved in the germination process [14,61]. However, intact fruits and hand-peeled stones did not differ significantly in the laboratory and both presented similar high germination rates under semi-natural conditions. Thus, we cannot make any solid conclusions about the inhibitory effects of the pulp in *P*. *mahaleb*, but maybe future germination tests using different concentrations of pulp extract might help to assess this effect. On the other hand, the removal or weakening of the endocarp through artificial scarification treatments was effective for enhancing germination response. Mechanical removal of the endocarp was more effective than chemical scarification. The same difference was reported in the native range of this species [45,46] and for other *Prunus* species [37,47]. Submerging the stones in sulfuric acid for 10 minutes reduced the mechanical resistance of the endocarp, enhancing the germination to similar values as those reported by Ghayyad *et al*. [45]. Longer periods of acid exposure have been reported to have irreversible negative effects on the germination rate of *P*. *mahaleb* [45,46] and also in other *Prunus* species [37,47].

### Disperser effects

In concordance with the literature, our results indicate that frugivores can show a variety of effects on the seed germination of an invasive fleshy-fruited plant [5]. Even birds that are close in terms of phylogenetic relationships and morphological characteristics, like the two *Patagioenas* species considered in the present work, produce extremely different effects on the fruits they consume, with one of them destroying almost all the seeds. The effect of dispersers was beneficial for germination under laboratory conditions, except in the case of two species, *P*. *maculosa* and *L*. *gymnocercus*. The higher germination response with respect to hand-peeled stones suggests that dispersers perform some kind of scarification process on the endocarp as well as pulp removal. The degree of erosion in the seed depends on the fruit and seed traits, and also on the disperser’s behavior, and morphological and physiological traits [14]. The length of the digestive tract, the presence of a muscular stomach, the composition of digestive fluids, water content, pH and other physiological factors, as well as the type of food ingested together with the fruit, affect the scarification process [15]. The passage through the digestive tracts of the dispersers and their differential retention times imply different exposures to stomach acids, leading to differences in the scarification process [62]. However, there is contradictory evidence about the effect of seed retention times on germination success [11,61–63].

We can distinguish two groups in the bird species we studied: those that regurgitate the stones and those that drop them through defecation. Regurgitators tend to process fruits quickly and drop them one at a time, whereas fruit-ingesting frugivores deposit seeds in fecal clumps after gut processing [15]. The stones individually regurgitated by *T*. *savana* were completely free of pulp vestiges and showed the highest germination rate, although they did not pass through the entire digestive tract. In contrast, *P*. *sulphuratus* regurgitates the stones in groups, among other food wastes, with higher proportions of pulp vestiges on their surface, which could be related to the lower germination rate with respect to that of *T*. *savana*. On the other hand, *M*. *saturninus* and *P*. *maculosa* defecated clumps of stones with higher levels of pulp retention than the regurgitated ones. While stones defecated by the former exhibit higher germination rates than hand-peeled stones, those defecated by the latter retained a higher proportion of pulp and did not show any significant differences with respect to both the control treatments. Seed regurgitation by Passerines generally occurs quickly (5–20 minutes), whereas defecated seeds are retained in the digestive tract for a longer time (20–90 minutes) [14,64]. The seeds that are regurgitated by certain species usually end up completely clean in spite of being retained less time in the digestive tract, in contrast to what happens with the seeds that are defecated, which commonly maintain adhered pulp vestiges [11,14].

Feces of the Pampas fox contained many intact fruits and stones with high amounts of adhered pulp vestiges, showing low germination values that did not differ from the control treatments. Mammals tend to deposit clumps with more seeds than do most birds, and their diet and faeces composition tend to be highly variable, affecting seed fate and seedling survival [15]. It is common to find intact fruits and pulp vestiges adhered to the seeds in the feces of some dispersers, with different consequences on the germination response [14]. We observed different degrees of pulp removal on the surface of stones among the dispersers and this seems to be reflected in the germination response. This is consistent with the idea that pulp has an inhibitory effect on germination. This association between pulp removal by frugivores and germination enhancement has been demonstrated for other fleshy-fruited invasive plants [5,11,62]. However, our results demonstrate that the differences in the germination responses of stones consumed by different dispersers cannot only be interpreted in the light of the degree of pulp removal, as scarification of the endocarp plays a main role.

Beyond the effects of vertebrate dispersers on the germination rate, the maximum germination rate recorded in the laboratory corresponded to stones treated by leaf-cutting ants, which deposit the stones totally free of pulp vestiges. By focal observations on some ant nests in the study area, we were able to see that many fruits were introduced into the nests. *Acromyrmex* ants concentrate high amounts of plant tissues in the nest, inside the growing chambers where fungi are cultivated to feed the larvae [65]. Worker ants can ingest part of the plant materials they transport (in this case, removing the fruit pulp and cleaning the stones). Moreover, contact with fungal mycelium inside the nest and exposure to its enzymes and acids might imply an important chemical scarification process [66]. Both processes might explain the high germination rate observed in our experiment. The material that is not degraded by the fungi, including *P*. *mahaleb* stones, is carried outside the nest, forming refuse dumps rich in organic matter, nutrients and water retention [65,67]. These refuse dumps create spots with adequate conditions for germination, and the establishment and survival of seedlings of different plant species, including exotic species [68].

### The environmental effect

The results we obtained under semi-natural conditions offer a supplementary interpretation to what we found in the laboratory. Even intact fruit showed seedling emergence in contrast to the null response obtained in laboratory. Robertson *et al*. [19] reported similar results where intact fruit showed much a lower germination percentage in the laboratory than in the field and they suggested this may be an artifact of the laboratory conditions, where leaching and microbial activity differ from the natural environment. In our experiment, the stones remained buried in the soil at a depth of 1 cm for several months until germination began. Soil microorganisms can contribute to pulp degradation, neutralizing its inhibitory effect and enabling germination even when stones are deposited with total or partial adhered pulp [20,69]. Similar results were obtained in other *Prunus* species, for which the pulp was degraded by soil microorganisms, enabling germination to occur in the field [20], whereas intact fruits did not germinate [50]. Jordaan *et al*. [11] carried out a greenhouse experiment with the seeds of fleshy-fruited invasive plants buried in the soil and postulated that germination occurred successfully after a few months due to pulp decomposition, despite the relatively low initial germination response of intact fruits. In our experiments, hand-peeled stones also showed an enhancement in seedling emergence under semi-natural conditions (55%) with respect to the germination response in the laboratory (11–15%). Moreover, all the stones that were consumed by dispersers showed an enhancement of 35–60% in their response under semi-natural conditions, in comparison to the laboratory test, even in the case of those treatments that were not significant in the laboratory. The soil microorganisms might have some effect on weakening the endocarp during the period between sowing and germination, together with the weathering effect of daily and seasonal temperature fluctuations, thereby facilitating the emergence of the embryo. Vivian-Smith and Gosper [21] demonstrated that seed burial at a depth of 1 cm enhanced seedling emergence of manually depulped fruits of three exotic *Asparagus* species in subtropical Australia, both under controlled irrigation and natural rainfall. This phenomenon could be of key importance for the fate of dispersed seeds under natural conditions.

Our tests in the experimental garden highlight the importance of soil processes, which supplement or replace the pulp removal and scarification processes that occur inside the dispersers’ digestive tracts, reducing the dependence of germination on this interaction [15,18,19]. Dispersers can transport seed to distances far away from the zone of influence of the parent plant, reducing the negative effects of density-dependent processes [17]. However, dispersed seeds are generally deposited on the ground where they are exposed to seed predators and environmental fluctuations, and post-dispersal processes at the deposition sites may be crucial for establishment [15,18,19]. Dispersed seeds might occasionally penetrate the soil if they become buried by post-dispersal events, even several months later. Moreover, secondary dispersers such as ants, small rodents and dung beetles, are well known for their roles in moving seeds beyond frugivore deposition sites to different specific micro-environmental conditions [5]. Therefore, it is important to consider post-dispersal processes, such as secondary dispersal or natural burial mechanisms, in order to understand the dispersal mechanisms of invasive fleshy-fruited plants which, as our results suggest, might be highly relevant to the seedling recruitment.

We did not record any loss in viability after the laboratory experiment. We also recorded seedling emergence during the second spring in the experimental garden trial. This suggests that most dispersed seeds might germinate during the first spring but that a certain proportion still remain dormant and maintain their ability to germinate in subsequent years, at least if they are exposed to adequate environmental conditions. On the other hand, the emerged seedlings showed a high survival rate during their first year. These processes might determine the dynamics of the invasion in the area.

## Conclusions

In previous studies, we proposed that *P*. *mahaleb* shows traits associated with successful invaders (small bright fruits, large crop size, gap-filling phenology) [30,41,70]. In particular, it constitutes a food resource that is not very common in the native flora, at least in the abundance with which it is offered. These aspects promote the attraction of several generalist dispersers. Our experiments exposed some of the main factors involved in the germination of *P*. *mahaleb* seeds and suggest that it benefits from a non-specialized treatment by generalist dispersers, which promote germination through pulp removal and endocarp scarification. Pulp removal only seems to have a weak effect on germination response, whereas endocarp scarification enhances germination success. The interaction between these two processes can explain the variability observed in the effects of dispersers. On the other hand, the high germination response under semi-natural conditions suggests that post-dispersal processes, like seed burial and exposure to natural conditions, could promote successful recruitment even in the case of fruits moved by dispersers that fail to enhance the germination response. This scenario highlights the need to consider the whole seed dispersal process and to combine laboratory and field tests. The high germination capacity of *P*. *mahaleb* under semi-natural conditions and the high seedling survival rates during their first year have important implications for the local community dynamics. The information obtained in studies like ours can contribute to a better understanding about the role of animals in invasive plant dispersal, enabling more effective modeling, prediction, invasion prevention and management.

## Supporting information

S1 Table Irrigation regime applied in the experimental garden trial. It shows the total amount of water used per month for *Prunus mahaleb* stones sown at *Jardín Botánico Pillahuincó*, Ernesto Tornquist Provincial Park.
(DOCX)

S1 Fig Images of stones treated by different dispersers. The pictures show different amounts of pulp vestiges adhered to the stone surface for *Prunus mahaleb* treated by different dispersers: *Acromyrmex lundi* (A), *Tyrannus savana* (B), *Mimus saturninus* (C), *Pitangus sulphuratus* (D), *Patagioenas maculosa* (E), *Lycalopex gymnocercus* (F), hand-peeled stones (G), intact fruits (H).
(TIF)

S1 File Data set of the germination test carried out under laboratory conditions. It contains the number of germinated seeds from a total of 20 initial seeds (Ngerminated), for each replicate (Replicate), for the different treatments considered (Treatment) and days after sowing (Days).
(CSV)

S2 File Data set of the germination test carried out in the experimental garden. It contains the occurrence of seedling emergence (Emergence, binary), for each individual seed initially sown (ID), for the different treatments considered (Treatment) and days after sowing (Days).
(CSV)

S3 File Data set of the percentage of pulp vestiges observed on stones treated by dispersers. It contains the estimated percentage of the stone surface that was covered with pulp vestiges (pulp.vestiges), using five categories (0%, under 25%, 25–50%, 50–75%, above 75%), for the different species (Species).
(CSV)
