# Supplementary material for: Generalist dispersers promote germination of an alien fleshy-fruited tree invading natural grasslands
Source: PLoS One. 2017 Feb 16;12(2):e0172423. doi: 10.1371/journal.pone.0172423 (PMC5312964; doi:10.1371/journal.pone.0172423)
Supplement: S1 Table — It shows the total amount of water used per month for Prunus mahaleb stones sown at Jardín Botánico Pillahuincó, Ernesto Tornquist Provincial Park. (DOCX) [file pone.0172423.s001.docx]

S1 Table. Irrigation regime applied in the experimental garden trial conducted with *Prunus mahaleb* stones at the Ernesto Tornquist Provincial Park, Argentina.

| **Month** | **Irrigation (l/m^2^)** | **Month** | **Irrigation (l/m^2^)** |
| --- | --- | --- | --- |
| APRIL | 74 | OCTOBER | 101.5 |
| MAY | 39 | NOVEMBER | 78 |
| JUNE | 20 | DECEMBER | 78 |
| JULY | 38 | JANUARY | 82 |
| AUGUST | 32.5 | FEBRUARY | 98 |
| SEPTEMBER | 69 | MARCH | 91 |

The quantity of water provided each month was equivalent to the median of monthly precipitation values corresponding to the historical records in the area for the period 1993-2009 (data provided by Ernesto Tornquist Provincial Park rangers and staff).
