# Supplementary figures and images for: Generalist dispersers promote germination of an alien fleshy-fruited tree invading natural grasslands
Source: PLoS One. 2017 Feb 16;12(2):e0172423. doi: 10.1371/journal.pone.0172423 (PMC5312964; doi:10.1371/journal.pone.0172423)

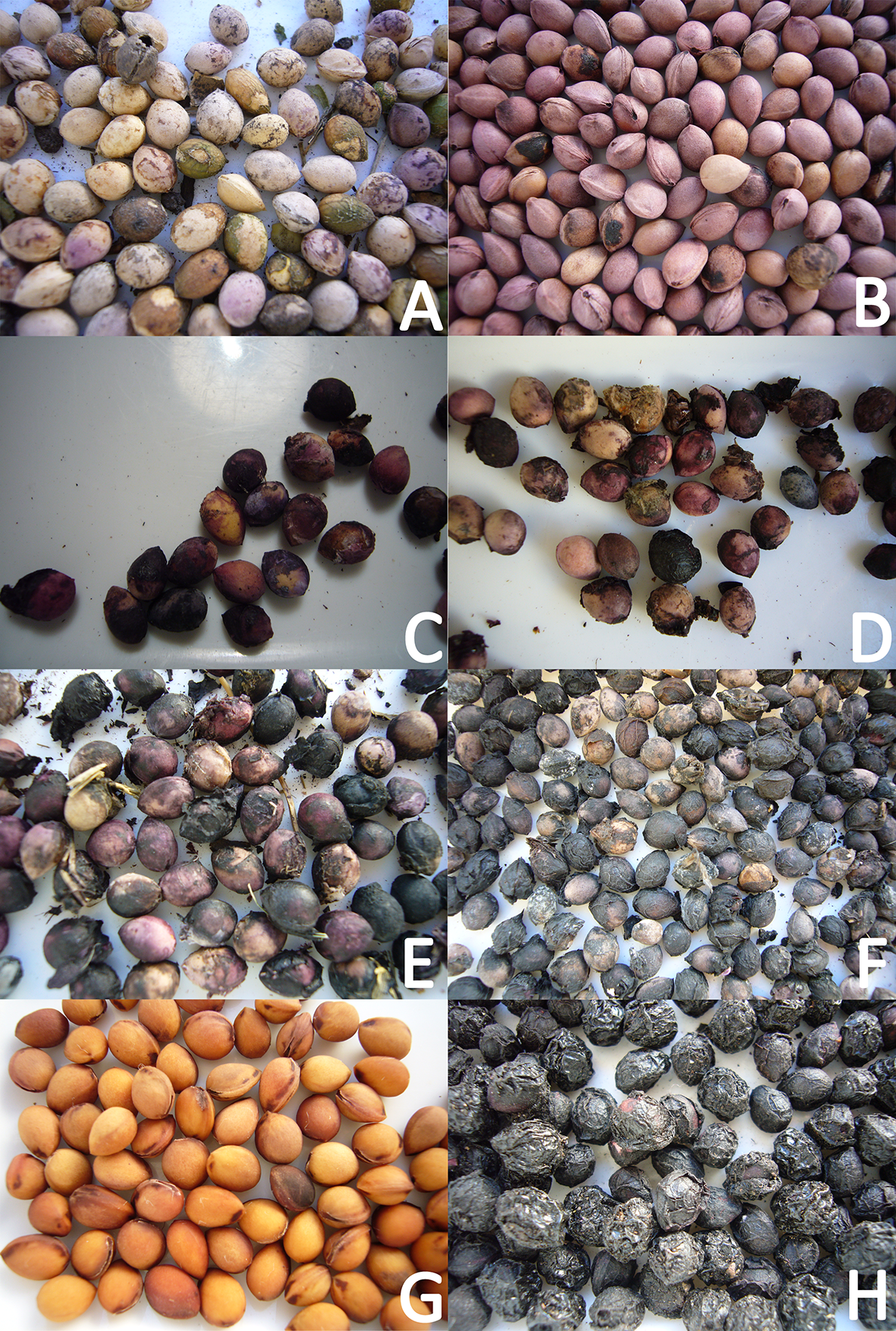

Supplement: S1 Fig — The pictures show different amounts of pulp vestiges adhered to the stone surface for Prunus mahaleb treated by different dispersers: Acromyrmex lundi (A), Tyrannus savana (B), Mimus saturninus (C), Pitangus sulphuratus (D), Patagioenas maculosa (E), Lycalopex gymnocercus (F), hand-peeled stones (G), intact fruits (H). (TIF) [file pone.0172423.s002.tif]
